# Case Report: Chronic Morel-Lavallée lesion: dynamic MRI progression, histopathological findings and surgical treatment

**DOI:** 10.3389/fmed.2026.1948713

**Published:** 2026-09-17

**Authors:** Bingyan Mao, Cheng Ma, Jicai Li, Zuoquan Qin, Shenke Xie

**Affiliations:** 1 Department of Orthopaedic Surgery, The Peopleʹs Hospital of Shimen County (Shimen Hospital of Changsha Medical University), Changde, Hunan, China; 2 Medical Imaging Center, The Peopleʹs Hospital of Shimen County (Shimen Hospital of Changsha Medical University), Changde, Hunan, China; 3 Pathology Department, The Peopleʹs Hospital of Shimen County (Shimen Hospital of Changsha Medical University), Changde, Hunan, China

**Keywords:** case report, chronic, closed degloving injury, Morel-Lavallée lesions, trauma

## Abstract

**Background:**

The Morel-Lavallée lesion (MLL) is a closed soft tissue avulsion injury resulting from shear force trauma. Chronic Morel-Lavallée lesions present significant management challenges due to the formation of fibrous caps, persistent fluid accumulation, and a tendency for recurrence. Nevertheless, the pathogenesis and imaging characteristics of these lesions remain poorly understood.

**Methods:**

This study presents a case of a chronic Morel-Lavallée lesion located in the right hip, with a disease progression lasting over 10 years. The MRI characteristics of the lesion were compared across different stages. The patient underwent open resection of the lesion, along with excision of excess skin and subcutaneous tissue, followed by negative-pressure drainage surgery. The efficacy of the treatment was evaluated through a 1-year postoperative follow-up.

**Results:**

MRI examinations at different time points revealed a progression of the lesion from a chronic organized hematoma (Mellado-Bencardino classification Type III) to a coexistence of a chronic organized hematoma (Type III) and subacute hematoma-like lesions (Type II), indicating dynamic progression rather than static stability of the chronic Morel-Lavallée lesion. Characteristic MRI findings included hematoma organization, fibrous capsule formation, round fat globules or fat septa, and layered fluid distribution within the cyst. Open surgery achieved complete resection of the lesion along with excess skin and subcutaneous tissue, followed by negative-pressure drainage, resulting in excellent wound healing; no recurrence was observed during the one-year follow-up, and hip joint function remained normal.

**Conclusion:**

The chronic progression of Morel-Lavallée lesions is a dynamic process characterized by MRI findings such as fat globules or fat septa, fluid level stratification, hematoma organization, and capsule formation, all of which aid in definitive diagnosis. For large, recurrent chronic Morel-Lavallée lesions, open surgical resection of the lesion, along with the excision of excess skin and subcutaneous tissue, combined with adequate negative pressure drainage, effectively eliminates dead space and reduces the risk of recurrence.

## Introduction

The Morel-Lavallée lesion (MLL) is a closed soft tissue avulsion injury caused by traumatic shear forces, commonly observed in high-energy trauma (1–4). Its core pathological feature is not merely hematoma formation; rather, it involves persistent fluid accumulation and local tissue necrosis triggered by mechanical separation. The clinical management of acute Morel-Lavallée lesions is well-established, including puncture drainage, pressure dressing, or surgical debridement (1, 3–8). However, inadequate early intervention or misdiagnosis may lead to fibrous encapsulation around the cavity, gradually transforming the lesion into a chronic, recurrent condition (9, 10), the mechanisms responsible for the persistence and progression of some chronic lesions remain incompletely understood. Additionally, the chronic inflammatory microenvironment may induce peripheral nerve compression, skin necrosis, or secondary infection, significantly complicating clinical management (2, 11–15). Currently, there are no unified diagnostic and therapeutic standards for chronic Morel-Lavallée lesions, and systematic research on their chronic progression mechanisms remains notably lacking. This article reports a case of a chronic Morel-Lavallée lesion persisting for over 10 years. By comparing MRI findings from different time points, it analyzes the key stages of progression in chronic Morel-Lavallée lesions and examines their characteristic MRI features, alongside a treatment approach that combines open surgical excision of the lesion with the removal of excess subcutaneous tissue, followed by negative-pressure drainage. The study aims to provide insights for the diagnosis and management of chronic Morel-Lavallée lesions in clinical practice.

## Case report

### Patient information and history

The patient is a 52-year-old male, measuring 165 cm in height and weighing 65 kg, resulting in a BMI of 23.9 kg/m^2^. For over 10 years, he has experienced swelling and limited mobility in the right hip due to an injury sustained in a traffic accident. A mass in the right hip has gradually enlarged, with swelling significantly worsening after physical activity; no medical treatment has been administered. The patient visited the outpatient department of our hospital on June 23, 2023. An MRI examination revealed a subcutaneous mass in the right hip, accompanied by hematoma and surrounding exudative lesions. While this condition cannot be definitively ruled out as being caused by post-traumatic vascular malformation, the patient declined surgical intervention. Six months ago, a significant increase in the size of the mass in the right hip was observed, along with worsening mobility limitations. Consequently, the patient was admitted to The Peopleʹs Hospital of Shimen County (Changde, China) on May 23, 2025, for further diagnosis and treatment. The patient denied any history of local skin trauma, infection, or other systemic diseases. This study is a retrospective case analysis conducted with the patient's informed consent and does not contain any personally identifying information. Data collection, diagnosis, and treatment complied with the principles of the Helsinki Declaration and were approved by the Medical Ethics Committee of The Peopleʹs Hospital of Shimen County (Approval No.: K2026001).

### Clinical findings

The physical examination upon admission revealed significant swelling in the right hip region, with no obvious redness, swelling, or ulceration on the skin surface. The skin temperature was normal, although skin tension was elevated, and no significant superficial vascular distension was observed. A large mass, measuring approximately 58 mm × 85 mm × 191 mm, was palpable subcutaneously. This mass was characterized by a firm consistency, well-defined borders, limited mobility, absence of significant fluctuance, and no marked tenderness. Movement of the right hip was restricted, while the blood supply, sensation, and motor function of the distal limb remained normal.

### Laboratory and imaging studies

The patient's blood, urine, and stool routine tests, along with coagulation function, liver and kidney function tests, thyroid and parathyroid function tests, tumor markers, and infectious disease screenings, all returned normal results. On June 23, 2023, an outpatient MRI revealed a large subcutaneous mass in the right hip, measuring 45 mm × 80 mm × 120 mm, characterized by heterogeneous signal intensity, irregular walls, and uniformly low signals at the margins. Within the mass, nodular and patchy areas of low signal intensity were observed, alongside regions of high T1-weighted signal intensity and patchy high T1-weighted signal intensity peripherally. On May 23, 2025, MRI demonstrated an increase in size of the mass, now measuring 55 mm × 83 mm × 181 mm, with well-defined borders, heterogeneous signal intensity, irregular walls, and low signals at the edges. The interior of the mass contained nodular and patchy areas of long T1-weighted signal intensity and hypointensity, while the lower part exhibited a cystic area of short T1-weighted signal intensity with hypointensity, which contained layered fluid levels (Figure 1).

**Figure 1:**
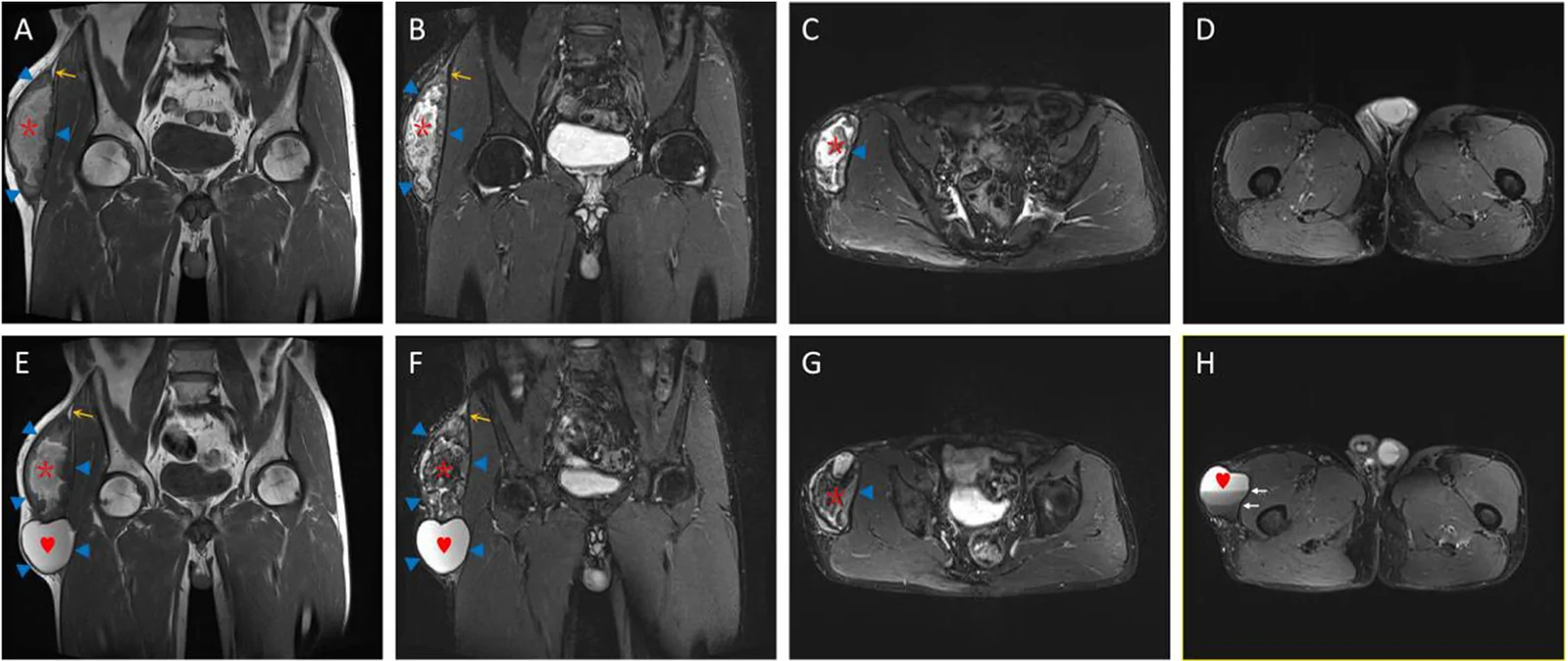
The patient underwent MRI examinations at 8 years **(A–D)** and 10 years **(E–H)** following right hip trauma. Images **(A)** and **(E)** display T1-weighted coronal views; images **(B)** and **(F)** illustrate T2-weighted fat-suppressed coronal views; and images **(C)**, **(D)**, **(G)**, and **(H)** present T2-weighted fat-suppressed sagittal views. The red asterisk (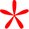) indicates a chronic organized hematoma (Mellado-Bencardino classification Type III), the red heart (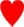) denotes subacute hematoma-like lesions (Mellado-Bencardino classification Type II), the blue triangle (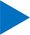) signifies capsular formation of the lesion, the white arrow (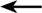) indicates fluid-level stratification, and the yellow arrow (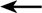) suggests possible fat septation.

### Treatment process and pathological results

The patient underwent resection of a right hip mass under continuous epidural anesthesia. During the procedure, the cyst wall, intracystic lesions, and excess skin and subcutaneous tissue were completely excised. To ensure that the tissues on both sides of the wound can be sutured under low tension and to minimize subcutaneous hematoma or fluid accumulation, the final distance between the remaining edges of the wound should be approximately 1 cm. Postoperatively, adequate drainage was achieved through two drainage tubes connected to a negative-pressure device (Negative pressure value: −125 mmHg). On postoperative day 4, the volume of negative-pressure drainage fluid was less than 10 mL; the drainage tube was then removed. The patient did not receive any antibiotics during hospitalization. Additionally, the patient's right hip joint movement was appropriately restricted during hospitalization to prevent intraoperative bleeding. The sutures were removed once wound healing was confirmed, and the patient was subsequently discharged. The postoperative pathological examination revealed that the right hip specimen consisted of a cystic benign lesion composed of fibrous connective tissue. The cyst wall exhibited fibrous hyperplasia and hyaline degeneration, along with infiltration of foam cells and inflammatory cells. Additionally, some areas of the cyst wall displayed multinucleated giant cell reactions, and extensive blood clots were aggregated and organized within the cyst (Figure 2).

**Figure 2:**
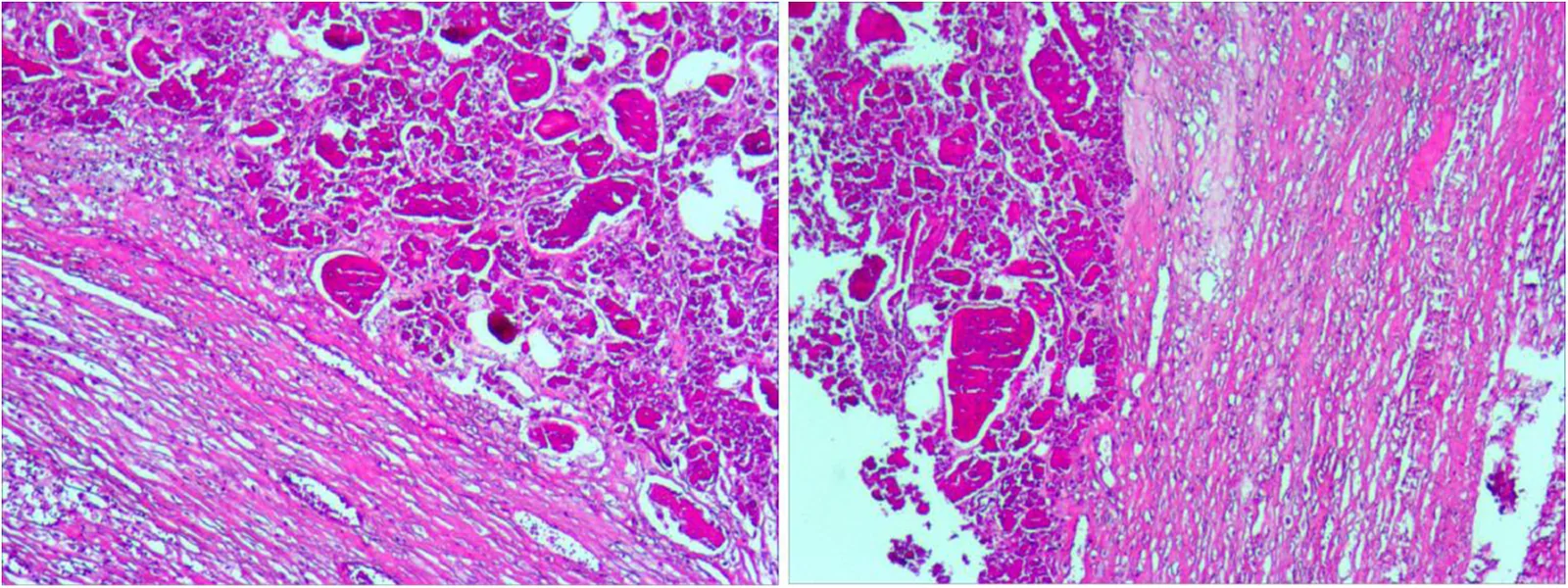
Postoperative pathological examination of the lesion revealed that the right hip specimen exhibited a cystic benign lesion composed of fibrous connective tissue. The cyst wall showed fibrous tissue hyperplasia and hyaline degeneration, with infiltration of foam cells and inflammatory cells within the wall. Some sections demonstrated multinucleated giant cell reactions, and extensive blood clots were observed organized within the cyst (HE staining, 20 × 10).

### Diagnosis and follow-up

The patient was ultimately diagnosed with a chronic Morel-Lavallée lesion of the right hip. At the 1-year follow-up, the condition remained stable, with no recurrence of masses in the right hip and normal hip joint function. Long-term outcomes necessitate continued monitoring. Additionally, we summarize the timeline of the patient's clinical events (Figure 3).

**Figure 3:**
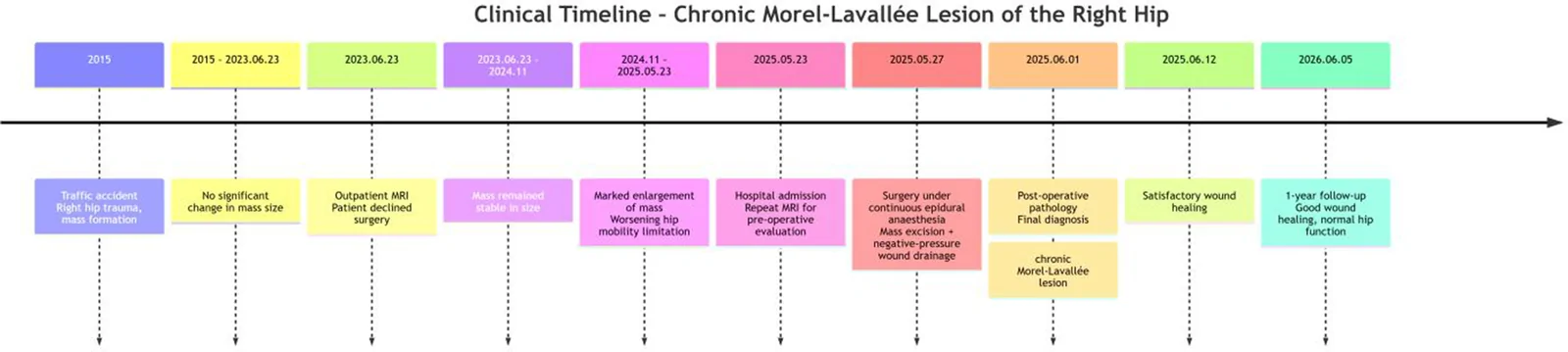
Detailed clinical course timeline from the patient's initial trauma in 2015 until the 1-year postoperative follow-up visit. Key events include: diagnostic imaging studies; the patient's initial refusal of surgical intervention; subsequent disease progression; surgical management involving open lesion resection combined with negative-pressure drainage; pathological confirmation; and a favorable healing outcome.

## Discussion

The Morel-Lavallée lesion (MLL) is a rare but clinically easily overlooked closed internal avulsion injury, first described and named by the French physician Victor-Auguste-François Morel-Lavallée. It is primarily caused by high-energy blunt trauma, such as traffic accidents or falls (16). Its pathogenesis involves shear forces that separate the subcutaneous tissue from the deep fascia, creating a potential cavity filled with blood, lymph fluid, and necrotic fat (1, 17). This lesion commonly occurs in the thigh, buttocks, and pelvis; however, the lumbar region, per knee area, and abdominal wall may also be affected (1, 3, 15, 17–21). This anatomical heterogeneity suggests that similar lesions can occur in any soft tissue region subjected to significant shear stress (9). Epidemiological data remain limited, as most evidence comes from case reports, single-center series, and retrospective studies of trauma populations (1). Early manifestations may include localized swelling, pain, fluctuance, or subcutaneous effusion. Due to intact skin surfaces, these findings are often mistaken for ordinary hematomas, leading to delayed diagnosis (14, 22). Ultrasound aids in initial screening (1, 2, 8, 23–26), while MRI provides enhanced visualization of lesion extent, fluid characteristics, and the presence of pseudocapsule formation (1, 4, 13, 20, 27, 28). The treatment of acute Morel-Lavallée lesions requires individualized approaches based on disease progression, lesion size, the presence of concurrent infections or fractures, and other factors. These treatment options include pressure dressing, aspiration, sclerotherapy, incision and drainage, and open debridement (4, 6, 7, 28). Studies indicate that up to 33% of adult cases may be missed during initial evaluation, particularly in polytrauma patients, where life-threatening injuries often mask local soft tissue manifestations, leading to diagnostic oversight (14, 29). Some patients do not seek medical attention until weeks to months after acute trauma, often due to localized fluctuating sensations, masses, pain, abnormal skin sensations, or recurrent fluid accumulation. As the condition progresses, the lesion may develop a fibrous capsule, evolving into a chronic lesion that leads to recurrent fluid accumulation, persistent pain, contour deformities, and increased risks of infection and fracture-related complications (10, 14, 15, 20, 30, 31). Thus, the formation of chronic Morel-Lavallée lesions results from the combined effects of delayed diagnosis and treatment, persistent cavity presence, and capsule formation. In this study, the patient was initially suspected of having a hematoma at an external hospital due to the early-stage injury. For an extended period following the injury, the mass in the right hip showed no significant growth, and the patient did not receive any medical intervention within tolerable limits. Ultimately, when the lesion in the right hip exhibited marked enlargement and continued progression, the patient was advised by physicians to undergo surgical intervention during hospitalization.

Current research typically classifies lesions with a duration of less than 3 weeks as acute, while those lasting longer than 3 weeks are categorized as chronic (19, 28). This simplistic temporal classification has limited practical value, as chronic lesions fundamentally reflect alterations in the biological behavior of the lesion rather than merely an extended duration. In some cases, even when the injury occurred before 3 weeks, MRI findings may reveal well-defined capsules, complex internal signal patterns, and recurrence after repeated aspiration; such lesions align more closely with chronic Morel-Lavallée lesions in terms of treatment decision-making. Conversely, smaller lesions with prolonged duration but lacking distinct capsules may still respond to conservative or minimally invasive management. There is a strong correlation between imaging findings and disease staging. MRI is widely recognized as a key tool for assessing lesion extent, fluid content, capsule formation, and relationships with surrounding structures. Mellado and Bencardino proposed the Morel-Lavallée lesion MRI classification system based on morphology, presence of a capsule, signal characteristics, and enhancement features (27, 32). This system defines six distinct types that broadly reflect varying stages and subtypes of lesions, ranging from simple serous effusions to hemorrhagic, organized, pseudocystic, or infectious complex lesions. The first three types are the most common: Type I represents a seroma, typically characterized by pure fluid accumulation, indicating an early or simple fluid injury, and exhibiting homogeneous low or high signals on T1 and T2-weighted images. Type II is a subacute hematoma with abundant blood components, resulting in more complex MRI signals, often showing layered fluid levels within the cyst, septal structures, or encapsulated fat globules, which reflect post-hemorrhagic progression. Type III denotes a chronic organized hematoma, suggesting prolonged disease duration with internal organization, septation, or gradual formation of a pseudocapsule, indicating chronic progression. Types IV–VI represent chronic atypical lesions. This classification aids in assessing lesion age, content composition, and degree of chronicity, providing a clinical basis for differential diagnosis and treatment planning, while also offering significant prognostic value for evaluating perioperative infection risk and selecting surgical timing (2, 3). When lesions occur near areas requiring open reduction and internal fixation, persistent fluid accumulation and poor skin or soft tissue condition may increase the risks of incision complications and deep infections.

Shear force serves as the initiating factor for Morel-Lavallée lesions, which fundamentally arise from inconsistent instantaneous or sustained displacement between the skin-subcutaneous tissue complex and the underlying deep fascia, muscles, or bony prominences. This displacement leads to the tearing of perforating vessels, capillary lymphatics, and fibrous septa. This injury pattern frequently occurs in anatomical regions characterized by high mobility of skin and soft tissues—such as the greater trochanter, pelvis, thigh, and per knee area—where deep supporting structures exhibit relatively limited mobility and stability. This indicates that the combined differences in local tissue compliance and bony prominence collectively determine susceptibility (7, 18, 30, 33–36). From a biomechanical perspective, tangential forces, rotational forces, or combined acceleration-deceleration stresses are more likely to cause superficial tissues to slide along the fascial surface. Subcutaneous fat lobules are interconnected by fibrous septa, while deeper layers are linked to the fascia via perforating structures. When external forces exceed the yield thresholds of these connections, detachment occurs along relatively low-resistance planes. Once such planes form, they create expandable potential cavities that provide an anatomical foundation for subsequent fluid accumulation (37).

Histologically, following shear injury, perforating arteries, veins, and lymphatic vessels rupture. At the site of the tear, hemorrhage occurs, and normal fluid return pathways are disrupted. Contusion and necrosis of adipose tissue further release cellular debris and lipid droplets, increasing the complexity of intraluminal contents, which typically include blood, lymph fluid, liquefied necrotic fat products, and inflammatory exudate. The acute phase is primarily characterized by hemorrhage due to vascular rupture; as capillary permeability increases and lymphatic drainage is interrupted, the chronic phase is dominated by protein-rich serous or serum-containing fluid. Elevated colloid osmotic pressure promotes further fluid retention while creating a microenvironment conducive to the recruitment of inflammatory cells and activation of fibroblasts. Under persistent inflammatory stimulation, the body attempts to encapsulate these substances, ultimately forming a fibrous capsule that surrounds the accumulated fluid and necrotic material, resulting in a chronic cystic structure. Thus, the chronic Morel-Lavallee lesion represents a locally initiated immunopathological process centered on chronic inflammation involving multiple immune cells (e.g., lymphocytes, histiocytes, macrophages/multinucleated giant cells), culminating in fibrous capsule formation and organization of chronic hematoma (3, 12, 20). Over time, changes in fluid composition may manifest as variations in MRI or puncture fluid appearance, ranging from homogeneous bloody discharge to layered fluid levels (3, 13, 20, 33, 37). Meanwhile, under shear forces, the torn ends of adipose tissue float within the hematoma. During the chronic phase, these fragments form oval-shaped lipid globules under the pressure of cystic fluid, remaining suspended in the liquid. In some cases, after partial tissue rupture, both ends connect to adjacent tissues, forming a fat septum. Alternatively, as the pathological fluid within the cyst decreases, the lipid globules are ultimately compressed toward the lesion margin, resulting in the formation of a fat septum.

Local pressure significantly influences the progression of lesions. When lesions are situated at the greater trochanter, buttocks, or lateral thigh, activities such as standing, walking, or lateral positioning can induce repeated mechanical stimulation. This stimulation leads to further microcirculatory damage and a redistribution of intracavitary fluid. Inappropriate external pressure or increased exudation due to infection may cause cystic fluid to spread peripherally, which results in tissue detachment and the subsequent rupture of arteriovenous perforating vessels and lymphatic vessels in the surrounding tissues. Therefore, the pathological outcome of chronic Morel-Lavallée lesions is not static but rather reflects a dynamic equilibrium maintained by the formation of fibrous capsules, mild inflammation, persistent exudation, and mechanical dead space. This balance elucidates why some lesions remain stable over time, while others may rapidly expand or recur following minor trauma, surgical intervention, or infection (Figure 4).

**Figure 4:**
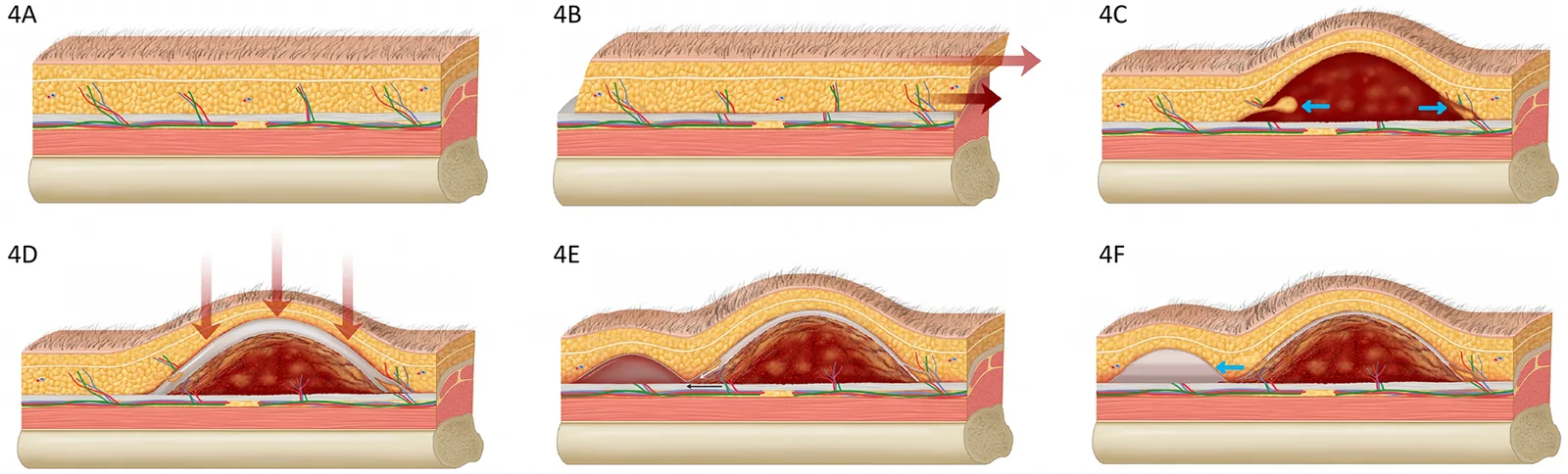
Formation mechanism diagram of chronic Morel-Lavallée lesion. Shear stress serves as the initiating factor; varying degrees of yield response to shear stress across different tissues lead to tearing of superficial fascial tissues and rupture of perforating blood vessels and lymphatic vessels **(A,B)**. Tissue tears between structures form an acute Morel-Lavallee lesion. One end of the torn adipose tissue is connected to the surrounding tissues, while the other end floats within the hematoma and forms fat globules under fluid compression. The two ends of the torn fat tissue are connected to the surrounding tissue, forming a fat septum **(C)**. 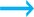 as indicated by the blue arrow. As the condition progresses chronically, hematoma organization occurs, cyst walls form, fat necrosis develops distally within the lipofascicular structures, and the cyst fluid expands into surrounding tissues under external pressure **(D)**. Cyst fluid expansion leads to rupture of new perforating vessels and lymphatic vessels, resulting in the formation of a new hematoma **(E)**. In mixed old lesions, new hematomas containing cystic fluid exhibit stratified liquid planes during deposition **(F)**. 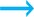 as indicated by the blue arrow.

In this study, the patient's initial outpatient MRI examination at our hospital revealed a right hip mass consistent with an organized hematoma accompanied by surrounding fluid accumulation, which meets the criteria for chronic organized hematoma (Type III) as outlined in the Mellado-Bencardino classification. The second MRI, conducted after hospitalization, demonstrated significant enlargement of the right hip lesion, along with the emergence of a heart-shaped cystic area exhibiting a short T1 hypointense fat-depleting signal distal to the original lesion. This finding indicates the coexistence of both chronic organized hematoma (Type III) and subacute hematoma-like lesions (Type II) according to the Mellado-Bencardino classification. This suggests that chronic Morel-Lavallée lesions are in a dynamic state of growth or progression rather than remaining static. Following the transition into the hematoma-organized phase (Type III), the residual cystic fluid between the organized mass and the cystic capsule continues to increase, leading to diffusion into surrounding tissue spaces under external forces. This results in the rupture of newly formed perforating blood vessels and lymphatic vessels, ultimately manifesting as subacute hematoma-like lesions (Type II) in subsequent MRI scans. In the early stages, fat tissue is violently torn by shear forces and floats within the hematoma. During chronic progression, under the pressure of intracavitary fluid, oval-shaped fat globules form and float within the cystic fluid. As the hematoma undergoes organization, these fat globules may be compressed toward the margins, leading to the formation of fat septa, which can be detected on MRI scans at various stages. In this study, fat septa were observed at the margins of the organized lesions in the patients’ MRI images. The composition of the intracavitary fluid also varies over time: blood, lymph fluid, and liquefied fat tissue exhibit distinct sedimentation patterns due to differences in density, resulting in a layered fluid distribution. On the second MRI examination, a distinct heart-shaped subacute hematoma-like lesion was clearly visible, with the intracavitary fluid presenting as three well-defined liquid planes. The uppermost layer primarily consists of liquefied fat and lymph fluid, while the lower two layers demonstrate significant low signal intensity on T2-weighted imaging due to hemosiderin, which is formed from the degradation of iron-containing hemoglobin in blood. To the best of the authors’ knowledge such unique cystic lesions with distinct fluid levels have not been previously reported in the literature. Therefore, we conclude that fat globules, fat septa, fluid stratification, hematoma organization, and capsule formation represent characteristic MRI manifestations of Morel-Lavallée lesions during chronic progression. At the same time, we believe the Mellado-Bencardino classification is primarily a morphological and MRI-based classification, and that the simultaneous presence of different imaging patterns may reflect components of different composition and/or age within the same lesion, without necessarily demonstrating a unidirectional biological sequence.

The treatment of chronic Morel-Lavallée lesions focuses on eliminating potential cavities associated with recurrent fluid accumulation, excising the formed fibrous capsule, and removing organized lesions. Once the lesion enters the chronic phase, it typically indicates the formation of an internal capsule, which impedes fluid reabsorption and increases the likelihood of recurrence following observation alone, local compression, or single-visit aspiration therapy. Therefore, the management of chronic Morel-Lavallée lesions should be tailored based on lesion size, disease duration, symptom severity, history of recurrence, presence of localized skin necrosis due to pressure, or concurrent infection. For small lesions with mild symptoms and minimal capsule formation, initial approaches may include compression dressing and close monitoring; some cases may also undergo ultrasound-guided puncture aspiration. However, for chronic lesions with prolonged duration, MRI evidence of capsule formation, large volume, recurrent fluid accumulation, functional or cosmetic impairment, or failure of conservative treatment, surgical intervention is generally preferred. This involves open debridement, excision of the fibrous capsule, thorough removal of blood, lymphatic fluid, and necrotic tissue, followed by closure fixation, negative-pressure drainage, or compression dressing to eliminate dead space and reduce recurrence rates. In this case, given the large size of the chronic Morel-Lavallée lesion and the limitations of minimally invasive surgery in removing excess skin and subcutaneous tissue due to lesion expansion resulting in residual cavities that predispose to recurrence (38), we opted for open surgery to excise the lesion along with excess skin and subcutaneous tissue, connecting two negative-pressure drainage tubes for adequate drainage (39). Postoperative wound healing was favorable, without significant skin necrosis or infection.

## Conclusion

We report a rare case of a chronic Morel-Lavallée lesion in the hip. Comparative analysis of MRI scans at different time points revealed that the chronic progression of this lesion is not static but rather a dynamic process. While MRI remains the gold standard for diagnosing chronic Morel-Lavallée lesions, characteristic imaging features include oval-shaped fat globules, layered intracystic fluid distribution, hematoma organization, and capsular formation during chronic development. To achieve thorough resection of organized lesions and cyst wall tissue while preventing recurrence due to excessive skin and subcutaneous tissue accumulation from prolonged chronic expansion, open excision combined with comprehensive negative-pressure drainage of the lesion and excess tissue. This treatment regimen proved successful in this patient case and may be considered for the management of specific types of large, chronic, encapsulated lesions. Finally, the mechanism underlying the progressive course of Morel-Lavallée lesions in this study was inferred based on imaging findings, histological examinations, and previous literature; this finding requires further investigation for confirmation.

## Data Availability

The original contributions presented in the study are included in the article/Supplementary Material, further inquiries can be directed to the corresponding author.
