# Supplementary material for: Case Report: Chronic Morel-Lavallée lesion: dynamic MRI progression, histopathological findings and surgical treatment
Source: Front Med (Lausanne). 2026 Sep 17;13:1948713. doi: 10.3389/fmed.2026.1948713 (PMC13626997; doi:10.3389/fmed.2026.1948713)
Supplement: Supplementary file 1 [file Datasheet1.pdf]

Supplementary Material

1 Supplementary Figures

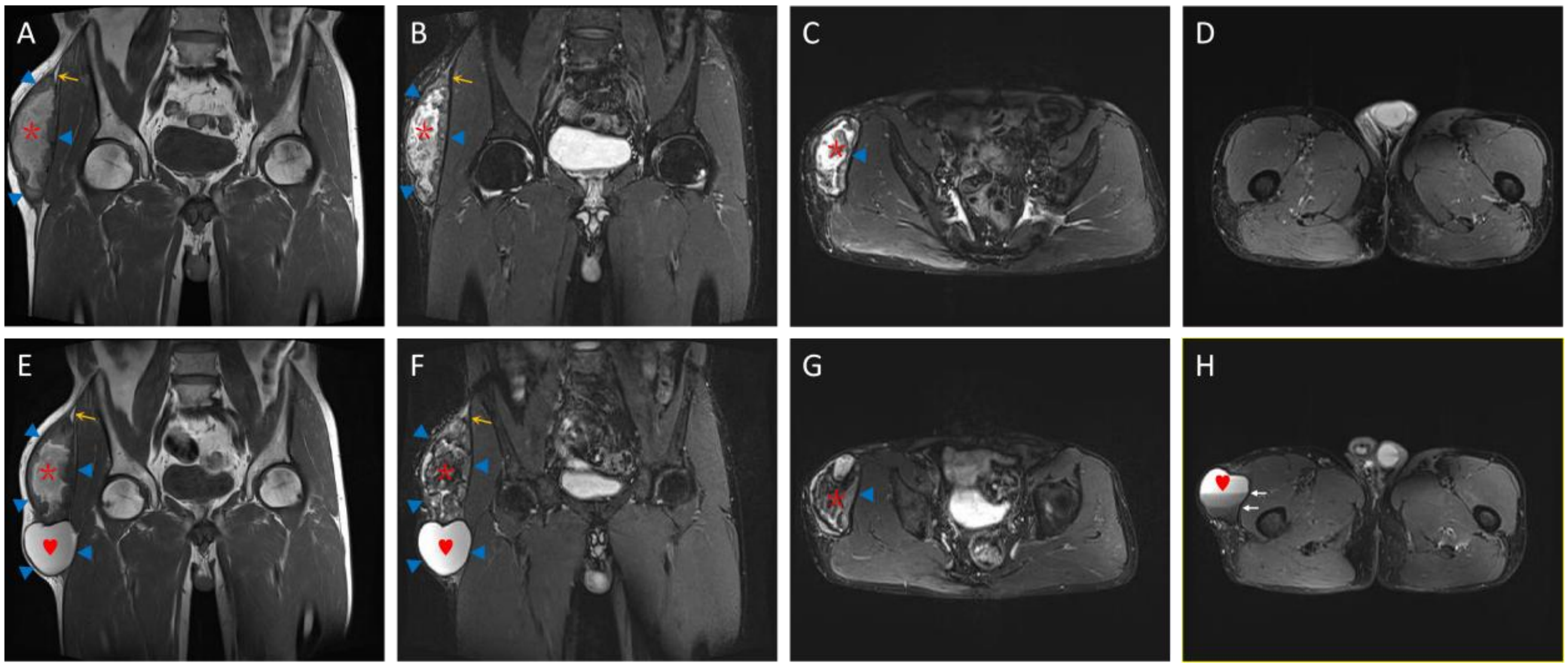

Figure 1. The patient underwent MRI examinations at 8 years (A – D) and 10 years (E – H) following right hip trauma. Images A and E display T1-weighted coronal views; images B and F illustrate T2-weighted fat-suppressed coronal views; and images C, D, G, and H present T2-weighted fat-suppressed sagittal views. The red asterisk (*) indicates a chronic organized hematoma (Mellado-Bencardino classification Type III), the red heart (♥) denotes subacute hematoma-like lesions (Mellado-Bencardino classification Type II), the blue triangle (▶) signifies capsular formation of the lesion, the white arrow (←) indicates fluid-level stratification, and the yellow arrow (←) suggests possible fat septation.

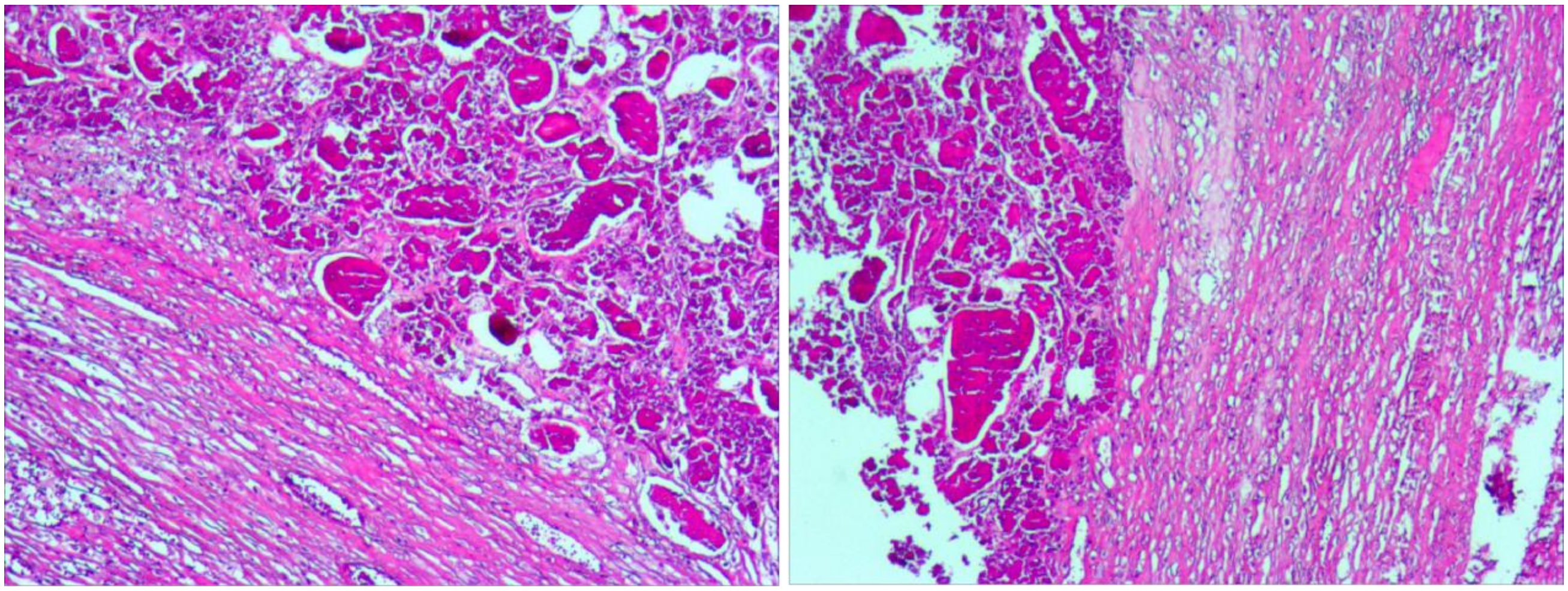

Figure 2. Postoperative pathological examination of the lesion revealed that the right hip specimen exhibited a cystic benign lesion composed of fibrous connective tissue. The cyst wall showed fibrous tissue hyperplasia and hyaline degeneration, with infiltration of foam cells and inflammatory cells within the wall. Some sections demonstrated multinucleated giant cell reactions, and extensive blood clots were observed organized within the cyst (HE staining, 20×10).

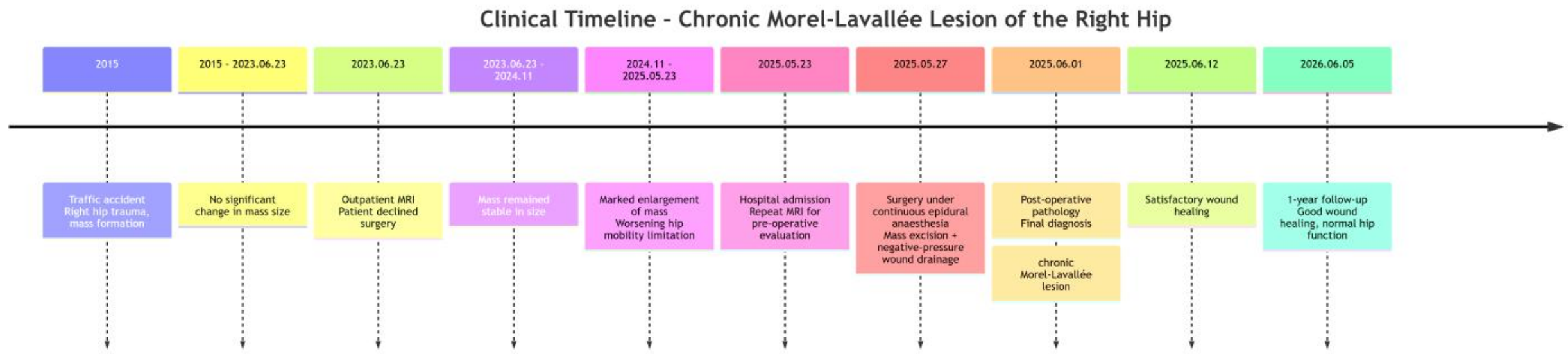

Figure 3.Detailed clinical course timeline from the patient's initial trauma in 2015 until the one-year postoperative follow-up visit. Key events include: diagnostic imaging studies; the patient's initial refusal of surgical intervention; subsequent disease progression; surgical management involving open lesion resection combined with negative-pressure drainage; pathological confirmation; and a favorable healing outcome.

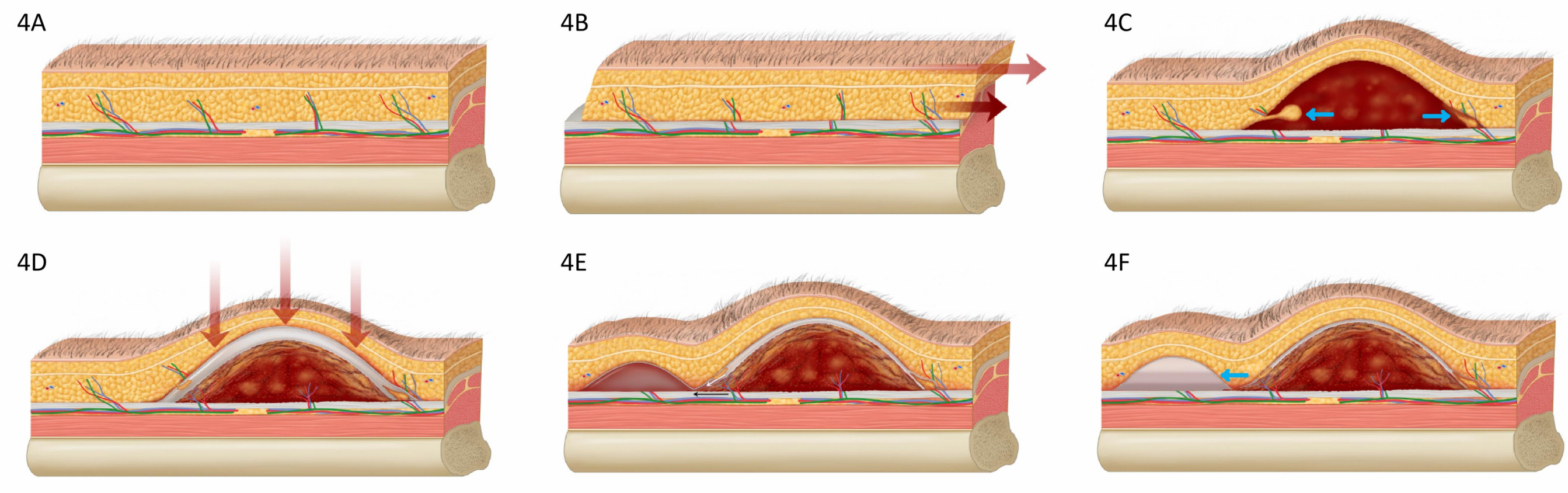

Figure 4. Formation Mechanism Diagram of Chronic Morel-Lavallée lesion.

Shear stress serves as the initiating factor; varying degrees of yield response to shear stress across different tissues lead to tearing of superficial fascial tissues and rupture of perforating blood vessels and lymphatic vessels (A, B). Tissue tears between structures form an acute Morel-Lavallee lesion.One end of the torn adipose tissue is connected to the surrounding tissues, while the other end floats within the hematoma and forms fat globules under fluid compression. The two ends of the torn fat tissue are connected to the surrounding tissue, forming a fat septum (C). → as indicated by the blue arrow. As the condition progresses chronically, hematoma organization occurs, cyst walls form, fat necrosis develops distally within the lipofascicular structures, and the cyst fluid expands into surrounding tissues under external pressure (D). Cyst fluid expansion leads to rupture of new perforating vessels and lymphatic vessels, resulting in the formation of a new hematoma (E). In mixed old lesions, new hematomas containing cystic fluid exhibit stratified liquid planes during deposition (F). → as indicated by the blue arrow.
